# Impacts of Orthognathic Surgery on Patient Satisfaction, Overall Quality of Life, and Oral Health-Related Quality of Life: A Systematic Literature Review

**DOI:** 10.1155/2019/2864216

**Published:** 2019-06-16

**Authors:** Rodrigo Zamboni, Flávio Renato Reis de Moura, Myrian Camara Brew, Elken Gomes Rivaldo, Marcylene Arruda Braz, Eduardo Grossmann, Caren Serra Bavaresco

**Affiliations:** ^1^Master of the Graduate Program in Dentistry, Lutheran University of Brazil (ULBRA), Canoas, Brazil; ^2^Postgraduate Program in Dentistry, Lutheran University of Brazil (ULBRA), Canoas, Brazil; ^3^Dentistry Course, Lutheran University of Brazil (ULBRA), Canoas, Brazil; ^4^Institute of Basic Medical Sciences, Federal University of Rio Grande do Sul (UFRGS), Porto Alegre, Brazil

## Abstract

Several treatments have been suggested to correct dentofacial abnormalities, including orthognathic surgery. The aim of the present systematic review was to assess the impact of orthognathic surgery on patient satisfaction, overall quality of life, quality of life related to oral health—and to orthognathic surgery in particular—among adult patients. Two investigators independently reviewed the available literature in the databases PubMed/MEDLINE, LILACS, SciELO, EMBASE, Trip, and Google Scholar (gray literature) based on the keywords “orthognathic surgery” and “quality of life.” An analysis of bias was performed based on the MINORS (methodological index for nonrandomized studies). A total of 245 relevant studies were retrieved from the databases, and 6 additional studies were located after a manual search of the references. Following selection based on titles, abstracts, and full-text analysis, 30 studies were included in the present systematic review. To evaluate quality of life before and after orthognathic surgery, 12 studies applied the surgery-related Orthognathic Quality of Life Questionnaire (OQLQ), 12 used the Oral Health Impact Profile (OHIP-14), and 4 used the Short Form Health Survey (SF-36). Orthognathic surgery results in improvements in quality of life both physically and psychosocially after surgery and is associated with high rates of patient satisfaction.

## 1. Introduction

Dentofacial deformities are characterized by disharmony among the face and dental bone structures, develop at a variable pace, and may have negative impacts on facial esthetics and stomatognathic system balance. In some cases, skeletal deformities are associated with malocclusion and an imbalance of the neuromuscular system, with consequent impairment of essential functions such as respiration, mastication, and phonation. In addition, the available evidence indicates negative effects related to self-esteem, self-confidence, and mental health [1, 2].

Several treatments have been suggested to correct dentofacial deformities. Orthognathic surgery combined with orthodontic treatment is considered the gold standard for correction of moderate-to-severe deformities [3]. Orthognathic surgery refers to surgical correction of the maxilla that affords proper alignment and positioning of the bones and teeth relative to the base of the skull. Combined with orthodontic treatment, orthognathic surgery provides adequate correction of malocclusion, especially for patients diagnosed with dentofacial deformity [4].

Depending on the severity of the problem, surgical correction varies from moving groups of teeth to complete repositioning of the mandible and maxilla. The aim of this treatment is to achieve functional occlusion, facial and dental symmetry, healthy orofacial structures, and stability between the dental arches [5]. In addition, some studies have found that dentofacial deformities not only affect the occlusal and functional aspects of the stomatognathic system but also impair the psychosocial and esthetic well-being of patients, i.e., all the components of quality of life [6].

Several studies have reported the impacts of orthognathic surgery on the psychological, social, physical, functional, and esthetic aspects of quality of life among patients both before and after surgery [6]. According to the World Health Organization (WHO), quality of life is defined as an individual's perception of his or her position in life in the context of the culture and value systems in which they live and in relation to their goals, expectations, standards, and concerns [7]. Despite the considerable increase in studies on the relationship between quality of life and oral surgery, a consensus regarding the best instrument to assess the outcomes of orthognathic surgery has not yet been reached [8].

Instruments for health measurement, such as the Short Form Health Survey (SF-36), assess impacts on general health status (not restricted to the orofacial area) [9]. Global oral health assessment instruments are used to investigate the impact of oral health on quality of life, such as the short form of the Oral Health Impact Profile (OHIP-14). Some instruments focus on specific situations, including the Orthognathic Quality of Life Questionnaire (OQLQ), which is widely used to investigate the impact of orthognathic surgery in the postoperative period [4].

In addition to the impact on quality of life, patient satisfaction in the postoperative period is another important outcome that should be evaluated, as it is one of the main goals of treatment. Kiyak et al. [10] observes that patients' expectations before surgery, and the information provided by the staff may be considered predictors of patient satisfaction after surgery. While the rate of satisfaction following orthognathic surgery is very high, some patients report dissatisfaction with the results despite a successful procedure. The reasons for such dissatisfaction and its impact on patient quality of life have not yet been fully elucidated [11, 12].

Given the aforementioned considerations, the aim of the present systematic review was to investigate the impacts of orthognathic surgery on satisfaction, overall quality of life, oral health-related quality of life, and orthognathic surgery-related quality of life among adult patients with dentofacial deformities as reported in observational and before-and-after intervention studies.

## 2. Methods

The present systematic review was registered at PROSPERO (CRD42018084936) and was conducted according to the quality criteria established in Preferred Reporting Items for Systematic Reviews and Meta-Analyses (PRISMA) [13].

Cross-sectional, case-control, longitudinal, and before-and-after intervention studies in which the main outcomes were patient satisfaction, overall quality of life, or oral health-related quality of life after orthognathic surgery were included for a qualitative review of the data.

Literature reviews, randomized clinical trials, pilot studies, studies without quality-of-life scores (missing data), and studies that did not describe the aforementioned outcome variables were excluded. Additionally, studies were excluded if they did not describe the mean global and domain scores on quality-of-life questionnaires, if they included patients with previous comorbidities with a potential impact on their quality of life, or if the main outcome was associated with orthodontic rather than surgical treatment. No restrictions were applied regarding the duration of the postoperative follow-up, the type of orthognathic surgery (maxilla, mandible, or both) or the type of dentofacial deformity. The search considered studies published in the past 50 years in English, Portuguese, or Spanish.

Two investigators (CSB and RZ) independently reviewed the available literature in the databases PubMed/MEDLINE, LILACS, SciELO, EMBASE, Trip, and Google Scholar (gray literature). A manual search of the references cited in the included publications was also performed.

The search strategy included the following keywords: “Orthognathic Surgery” (MeSH Terms) OR “Orthognathic Surgical Procedures” (MeSH Terms) OR “Orthognathic Surgeries” OR “Surgeries, Orthognathic” OR “Surgery, Orthognathic” OR “Maxillofacial Orthognathic Surgery” OR “Jaw Surgery” OR “Orthognathic Surgery, Maxillofacial” OR “Surgeries, Maxillofacial Orthognathic” OR “Surgery, Maxillofacial Orthognathic” AND “Quality of Life” (MeSH Terms) OR “Life Quality” OR “Health-related Quality Of Life” OR “Health-related Quality of Life.”

Titles and abstracts were independently analyzed by both reviewers to screen for potentially eligible studies for inclusion in the systematic review. The reviewers reached a consensus regarding the articles to be subjected to full-text analysis for potential inclusion in the systematic review.

The two reviewers independently collected and entered relevant information in a spreadsheet specifically designed for data collection. In cases of disagreement, a third, more experienced reviewer would be called. The collected data included the publication year, author's name, country, study design, number of participants, type of surgery, methods for data collection, duration of follow-up, and results. The results for patient satisfaction, overall quality of life (SF-36), oral health-related quality of life (OHIP-14), and orthognathic surgery-related quality of life (OQLQ) are presented in individual tables, which include the mean and standard deviation of the statistically significant data. The primary outcomes were as follows:

- Patient satisfaction
- Overall quality of life
- Oral health-related quality of life after orthognathic surgery.

The included studies were analyzed based on the MINORS (the methodological index for nonrandomized studies) [14]. The following sources of potential bias were considered “a clearly stated aim; inclusion of consecutive participants; prospective collection of data; endpoints appropriate for the aim of the study; unbiased evaluation of the study endpoints; a follow-up period appropriate for the aim of the study; loss to follow-up of less than 5%; and prospective calculation of the sample size” [14]. For studies including comparisons between groups, the following items were considered: an adequate control group; contemporary groups; the baseline equivalence of groups; and adequate statistical analyses. Scores were assigned as follows: 0 (not reported); 1 (reported but inadequate); and 2 (reported and adequate). The ideal global score is 16 for noncomparative studies and 24 for comparative studies.

The meta-analysis could not be performed for any assessment time point due to inconsistencies among the studies [15].

## 3. Results

**Figure 1:**
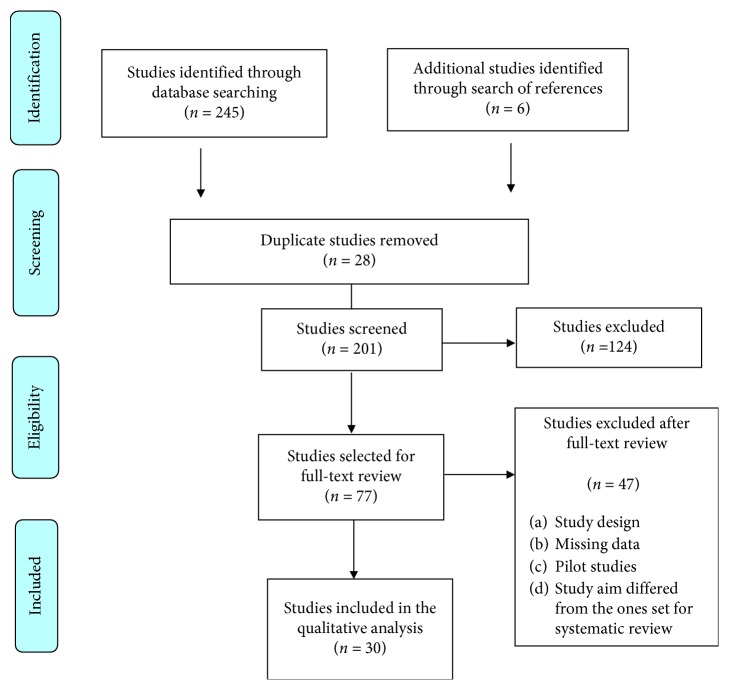
Flow chart for article selection.

The initial search identified 245 relevant studies in the aforementioned databases. Six additional studies were identified after a manual search of the references cited in the included articles. After analysis of the titles, abstracts, and the full texts of the articles, 30 studies were selected for the present systematic review, including a total of 1,510 patients. The study selection flow chart can be observed in Figure 1.

The retrieved studies exhibited wide variability in terms of study design, follow-up duration, and instruments used to measure quality of life. Additionally, the countries where the studies were conducted varied considerably, including countries from Europe, North, Central, and South America, and Asia and the Middle East. The main surgical procedures used were Le Fort I osteotomy and mandibular bilateral sagittal split osteotomy. Qualitative descriptions of the studies included in the present review are provided in Table 1.

Ten studies assessed patient satisfaction after orthognathic surgery. Among the validated questionnaires applied, the visual analogue scale (VAS) and Patient Satisfaction Questionnaire (PSQ) predominated. The VAS consists of a 10-centimeter line with well-defined ends: the left end represents “no problems” and the left end represents “major problems.” The PSQ contains four sections (involvement in clinical planning, surgical intervention, immediate postoperative care, and late postoperative follow-up) with responses to items measured on a 7-point Likert scale. Descriptions of the studies assessing patient satisfaction after orthognathic surgery are provided in Table 2.

The satisfaction rates reported in the studies were high, exceeding 85% when the patients who reported being very satisfied or satisfied were combined. Dissatisfaction was related to the occurrence of postoperative complications, information before surgery, unrealistic expectations regarding postoperative discomfort and recovery, weight loss, psychological changes before and after surgery, neuroticism, and external motivation [24, 25].

Dissatisfaction was reported in two studies, with a rate of approximately 7.5–8%, which tended to decrease throughout the follow-up. One of these studies compared satisfaction between patients subjected to the surgery-first approach and those subjected to the conventional orthodontic-first approach but did not detect a significant difference [38].

To assess quality of life before and after orthognathic surgery, 12 studies applied the OQLQ, 11 used the OHIP-14, and 5 used the SF-36. Other validated questionnaires were also used, such as the World Health Organization Quality of Life-Bref (WHOQOL-Bref) [21] and the Sense of Coherence 29-item scale (SOC-29) [40].

The OQLQ includes 22 questions distributed across four domains: facial esthetics, oral function, awareness of facial esthetics, and social aspects related to dentofacial deformity. Items are scored on a Likert scale ranging from 1 (it bothers you a little) to 4 (it bothers you a lot). The score for each domain is obtained by summing the scores given to the corresponding items; lower scores denote a lower impact on quality of life.

All the included articles reported improved OQLQ global and domain scores after surgery. The follow-up duration ranged from 1 to 21 months. Two studies compared the surgery-first approach to conventional orthognathic surgery (orthodontic treatment before surgery). Orthodontic treatment before surgery significantly increased the OQLQ scores, and the scores decreased again after surgery (*p* < 0.001) [32, 39]. The greatest impacts of surgery were in the domains facial esthetics, oral function, and social aspects [4, 23]. The studies that applied the OQLQ are described in Table 3.

The OHIP is used to assess negative outcomes in three dimensions—social, psychological, and physical—of the seven dimensions of quality of life proposed by Patrick and Bergner [41]. The OHIP also assesses changes related to oral health status in general rather than effects attributable to specific oral disorders. All impacts on the OHIP are rated as adverse results; therefore, it does not measure favorable oral health aspects. The full version of the OHIP contains 49 questions, while the shorter version contains 14 questions. Five responses are possible for each question: “very often,” “often,” “occasionally,” “hardly ever,” and “never,” which are scored as follows: 0 (never or not applicable), 1 (hardly ever), 2 (occasionally), 3 (often), and 4 (very often). A lower the score corresponds to a weaker negative impact of an intervention on quality of life [42].

The present review included 11 studies that applied the OHIP-14 to assess quality of life after surgery, as described in Table 4. The global score and all domain scores decreased after surgery in a time-dependent manner. Two articles reported increased scores 6 weeks [9] and 1 month after surgery [30]. In the assessment per surgical procedure, bimaxillary osteotomy combined with genioplasty resulted in better scores for the domains dissatisfaction with esthetics and psychological discomfort compared to bimaxillary osteotomy alone [27]. However, a significant difference was not found in the scores obtained in the late postoperative period between the groups. Quality of life improved in both groups after orthognathic surgery independent of the type of dentofacial deformity (Class II or III) [39].

The SF-36 includes one question to compare an individual's general state of health with that of 1 year ago and 35 items divided into 10 questions to investigate the individual's perception of their health status in the *previous* 4 weeks. The questionnaire includes eight domains categorized as either physical (physical functioning, physical role functioning, bodily pain, and general health perceptions) or mental (mental health, emotional role functioning, social role functioning, and vitality). Responses are scored from 0 to 100, corresponding to the poorest and best situations, respectively.

Four of the included studies used the SF-36 to assess the impact of orthognathic surgery on quality of life (Table 5). Overall quality of life improved after orthognathic surgery, especially for the component physical health. The scores for the domains mental health, vitality, and social role functioning increased in the late postoperative period compared to those in the presurgery period.

The results of the bias analysis of all the included studies based on the MINORS is described in Table 6. None of the studies described the sample size calculation, which may have influenced their external validity. None of the studies mentioned blinding during data analyses. Because participants communicate their responses directly to the professionals in charge of their follow-up, the possibility of overestimation of favorable responses cannot be ruled out. Another relevant point is the high rates of losses, which may have influenced the results.

## 4. Discussion

Substantial attention has been directed toward understanding health outcomes among patients in terms of their well-being in the past decades, with consideration for the concept of viewing patients as a whole, and they should be appraised from both physical and behavioral perspectives [30]. Regarding individuals with dentofacial deformities, the earliest studies, which were conducted in the 1980s, reported higher rates of negative self-perception among participants with a marked overjet or deep bite than among participants with normal occlusion, mainly in association with esthetic and functional limitations. In addition, body image concerns were substantially more frequent among women with malocclusions [43, 44]. Within this context, orthognathic surgery emerged as a strategy to modify the relationship between the maxilla and the mandible, leading to dramatic changes in the quality of life of patients with dentofacial deformities.

All the studies included in the present systematic review reported high rates of patient satisfaction and improved oral health-related quality of life after orthognathic surgery. However, the analysis of bias identified consistent weaknesses mainly related to sample size calculation and blinding during data analyses. The small sample size in many studies may substantially impair the external validity of the data.

Another possible source of bias is data collection after surgery. One may expect that a longer interval since surgery corresponds to better results for patient satisfaction. In addition, data collected in retrospective studies may not be reliable due to memory bias, thus requiring metaregression of the data in the analysis of impacts on outcomes.

Some evidence indicates that satisfaction after orthognathic surgery is directly related to the information provided by professionals before surgery regarding possible limitations and difficulties related to the surgical procedure [16] (MINORS 6). Postoperative complications, such as paresthesia, edema, pain, mastication difficulties, and limited mouth opening, have been described as modifiers of quality-of-life scores. Corso et al. [30] (MINORS 13) found poorer OHIP-14 scores 1 month after surgery, probably due to more severe postoperative complications.

The study performed by Kurabe et al. [36] (MINORS 10) with the questionnaire OHIP-54, which includes five additional questions on the temporomandibular joint (TMJ), detected significant increases in scores before and after surgery among patients with TMJ symptoms and/or limited mouth opening compared to those among asymptomatic patients. However, these authors did not find a significant difference in quality-of-life scores among patients with postoperative lower lip or chin paresthesia. According to Murphy et al. [4] (MINORS 10), the main reasons for dissatisfaction are related to the duration of treatment and eventual cancellation of surgery.

Favorable outcomes related to self-concept and social interactions before and after surgery seem to be associated with patient satisfaction and improvements in quality-of-life indicators [36] (MINORS 10). Therefore, satisfaction with surgery is not exclusively associated with the surgeon's ability but also with the physical and psychological aspects of patients.

Regarding questionnaire selection, some studies suggest that generic instruments for quality-of-life assessment, such as the SF-36, have poor sensitivity to detect changes in oral health or limited final construct validity. Therefore, questionnaires specific to certain conditions or diseases are needed [25] (MINORS 10). The present review detected predominant use of the OHIP-14 and OQLQ among the analyzed studies, which exhibit higher sensitivity for detecting the impact of orthognathic surgery on the quality of life of patients.

Significant gender differences were not found in OHIP-14 or OHIPJ-54 scores [25] (MINORS 10), [36] (MINORS 10). Corso et al. [30] (MINORS 13) reported that the rates of negative impacts on quality of life before (*p*=0.01), 1 month (*p*=0.038), and 3 months (*p*=0.025) after orthognathic surgery were higher among women. These differences may be related to cultural elements inherent to the setting where the study was conducted.

Kurabe et al. [36] (MINORS 10) asserted that oral health-related quality-of-life scores tend to be poorer among older versus younger patients. Based on the studies included in the present systematic review, age (which ranged from 20 to 40 years old) did not seem to have a direct impact on outcomes, as all studies detected improvement in the participants' quality of life.

Analysis of the variable “type of facial deformity” has paramount importance. Significant differences were not found in OHIP-14 scores according to the type of malocclusion [30]. However, Baherimoghaddam et al. [33] (MINORS 18) found significant differences in OHIP-14 scores between patients with Class II and those with Class III malocclusion. A significant difference was not found in the late perioperative period between patients undergoing the surgery-first approach or conventional treatment.

A discussion on cultural aspects related to quality of life is necessary. Abdullah [31] (MINORS 7) observed that the mean scores obtained in his study, which was conducted in Saudi Arabia, were higher than those reported by Lee et al. [14] (MINORS 12) for a Chinese population. Given the conservative and intimate nature of Saudi society, Abdullah [31] (MINORS 7) believes that the participants in his study were more sensitive to others' opinions about their appearance and behavior. Interestingly, as shown in Table 1, none of the included studies were performed with African populations, and very few were conducted in South America. The socioeconomic and cultural characteristics of such populations should be considered in order to fully comprehend the impact of orthognathic surgery on their quality of life, the absence of which may reflect a possible publication bias.

The studies included in the present systematic review investigated two techniques to treat dentofacial abnormalities: the orthodontic/orthognathic treatment combination, in which orthodontic treatment is performed before surgery, and the surgery-first approach, in which orthodontic treatment is performed after surgery. Favorable OHIP-14 outcomes were obtained when genioplasty was combined with bimaxillary osteotomy for prognathic women. Therefore, the type of surgical procedure may impact the quality of life of patients [27] (MINORS 14).

Regarding clinical correlations, which may be attributed to significant changes in quality-of-life scores and clinical parameters, Rustemeyer and Gregersen [25] (MINORS 10) found that larger cephalometric changes in the mentolabial angle corresponded to greater changes in OHIP-14 scores for the domains functional limitation (*r* = 0.527), physical pain (*r* = 0.831), psychological discomfort (*r* = 0.530/0.598), physical disability (*r* = 0.480), and social disability (*r* = 0.504).

Reductions in the SNB angle, facial convexity angle, and lower lip protrusion exhibited negative correlations with painful aching, the feeling of embarrassment, and difficulty relaxing. According to the authors, these negative correlations seemed to be related to the time required for some patients (approximately 30%) to adapt to a new facial contour, which was up to 24 months after surgery. An alternative explanation for this finding may be the small sample size, which may have resulted in data with high levels of bias. Therefore, the existence of direct relationships between changes in quality-of-life scores and objective clinical parameters that can likely predict outcomes remains inconclusive.

Based on the existing studies regarding orthognathic surgery-related quality of life, several concerns remain in terms of surgical methods, the amount of bone displacement during surgery and standardization of assessment time points after surgery. More controlled studies are recommended to achieve a better understanding of the effects of these factors on quality-of-life scores.

## Figures and Tables

**Table 1:** Qualitative descriptions of the included studies (*n*=30).

Authors/year	Study design	Country	Sample size	Type of orthognathic surgery	Methods for collection of data regarding the outcomes satisfaction and quality of life
(1) Cunningham et al. [16]	Retrospective (postoperative analysis) Prospective (preoperative analysis)	United Kingdom	100 patients (postoperative analysis) 83 patients (preoperative analysis)	Not reported	(1) Satisfaction: structured questionnaire developed by the authors with ranked responses (very satisfied, moderately satisfied, dissatisfied, very dissatisfied) (2) Self-esteem: Rosenberg Self-Esteem Scale (3) Depression scale
(2) Forssell et al. [17]	Prospective	Finland	Initial sample: 104 patients Final sample: 31 patients responded to the postoperative questionnaire	Mandibular sagittal split osteotomy (80 patients); Le Fort I maxillary osteotomy (6 patients); bimaxillary osteotomy (14 patients)	(1) Visual analogue scale (VAS): satisfaction with the results (2) Questionnaire for assessment of psychological well-being on a Likert scale (7 points)
(3) Bertolini et al. [18]	Prospective	Italy	20 patients	Not reported	(1) Satisfaction: structured questionnaire developed by the authors with ranked responses (very satisfied, moderately satisfied, dissatisfied, and very dissatisfied) after surgery (2) Minnesota multiphasic personality Inventory (3) Anxiety: State-trait anxiety inventory (STAI) (4) Depression: Zung Self-rating anxiety Scale
(4) Busby et al. [19]	Retrospective	USA	79 patients	Mandibular ramus osteotomy; maxillary advancement; combination of both procedures	(1) Satisfaction: 25-item questionnaire to assess satisfaction with postoperative changes, preoperative perception and overall satisfaction with the surgery (2) Perception of function and occlusion (3) Problems with facial sensations (4) Postoperative perceptions
(5) Lee et al. [9]	Prospective	Japan	36 patients	Bimaxillary osteotomy	(1) SF-36 (2) OHIP-14 (3) OQLQ
(6) Al-Ahmad et al. [20]	Retrospective	Jordan	136 patients (35 patients in the postsurgery group)	Not reported	(1) OQLQ (2) SF-36
(7) Choi et al. [3]	Prospective	Japan	60 patients	Bimaxillary osteotomy	(1) SF-36 (2) OHIP-14 (3) OQLQ
(8) Silva et al. [21]	Prospective	Brazil	15 patients	Bimaxillary osteotomy; mandibular setback and maxillary advancement	(1) WHOQOL-Bref
(9) Rustemeyer et al. [22]	Prospective	Germany	50 patients	Bimaxillary osteotomy	(1) OHIP-14
(10) Khadka et al. [23]	Prospective	China	Total: 158 patients Group A (orthodontics/orthognathic): 115 patients Group B (immediate surgical correction): 43 patients	Group A: sagittal osteotomy; intraoral vertical ramus osteotomy; Le fort I osteotomy; mandibular anterior segmental osteotomy Group B: mandibular osteotomy; L-shaped zygomatic osteotomy	(1) SF–36 (2) OQLQ
(11) Murphy et al. [4]	Prospective	Ireland	Initial sample: 62 patients Final sample: 52 patients	Bimaxillary osteotomy, mandibular setback	(1) OQLQ (2) VAS (3) GTS: Global transition Scale
(12) Khattak et al. [24]	Retrospective	United Kingdom	135 patients	Maxillary advancement and mandibular setback; bimaxillary advancement; condylectomy; maxillary posterior impaction; maxillary distraction osteogenesis; mandibular anterior segmental osteotomy	(1) PSQ
(13) Rustemeyer and Gregersen [25]	Prospective	Germany	30 patients	Bilateral sagittal split osteotomy of the mandibular ramus	(1) OHIP-14
(14) Trovik et al. [26]	Retrospective	Norway	Initial sample: 78 patients Final sample: 36 patients	Bilateral sagittal split osteotomy for mandibular advancement	(1) VAS (2) OIDP
(15) Rustemeyer and Lehmann [27]	Retrospective	Germany	Sample total: 60 patients Group bimaxillary osteotomy: 30 patients Group bimaxillary osteotomy with genioplasty: 30 patients	Bimaxillary osteotomy with or without genioplasty	(1) OHIP-14
(16) Wee and Poon [28]	Retrospective	Singapore	Initial sample: 114 patients Final sample: 41 patients	Le fort I osteotomy and/or mandibular bilateral sagittal split osteotomy	(1) OQLQ (2) OHIP-14
(17) Goelzer et al. [5]	Prospective	Brazil	74 patients	Not reported	(1) OHIP-14
(18) Schwitzer et al. [29]	Prospective	USA	Total sample: 49 patients Matched samples: 16 patients	Le fort I osteotomy and/or mandibular bilateral sagittal split osteotomy	(1) FACE-Q
(19) Corso et al. [30]	Prospective	Brazil	Control group: 60 patients Surgery group: 30 patients	Not reported	(1) OHIP-14
(20) Abdullah [31]	Retrospective	Saudi Arabia	17 patients	Mandibular, maxillary or bimaxillary osteotomy	(1) OQLQ
(21) Park et al. [32]	Prospective	South Korea	Initial sample: 44 patients Final sample: (a) Conventional surgery group: 15 patients (b) Surgery-first group: 11 patients	Bilateral sagittal split osteotomy of the mandibular ramus; Le fort I osteotomy	(1) OQLQ
(22) Baherimoghaddam et al. [33]	Prospective	Iran	Initial sample: 75 patients Final sample: 58 patients Group class II: 28 patients Group class III: 30 patients	Le fort I osteotomy; bilateral sagittal split osteotomy of the mandibular ramus	(1) OHIP-14
(23) Kilinc and Ertas [34]	Retrospective	Turkey	Total sample: 60 patients Control group: 30 class I patients Test group: 30 class II patients	Maxillary advancement, mandibular setback or both procedures and genioplasty	(1) OQLQ (2) OHIP-14 (3) SF-32
(24) Silva et al. [35]	Prospective	Sweden	Initial sample: 55 patients Final sample: 50 patients	Le fort I osteotomy; bilateral sagittal split osteotomy of the mandibular ramus	(1) OHIP-14 (2) OQLQ
(25) Kurabe et al. [36]	Retrospective	Japan	Surgery group: 65 patients Control group: 14 patients with class I occlusion	Le fort I osteotomy; bilateral sagittal split osteotomy of the mandibular ramus	(1) OHIPJ-54
(26) Bogusiak et al. [37]	Retrospective	Poland	Total sample: 90 patients Final sample: 66 patients	Bilateral vertical ramus osteotomy by the external approach; extraoral vertical ramus osteotomy (EVRO); bilateral sagittal split osteotomy of the mandibular ramus by the internal approach; bimaxillary osteotomy	(1) Satisfaction with life scale: SAT
(27) Huang et al. [38]	Prospective	China	Total sample: 50 patients Surgery-first group: 25 patients Conventional treatment group: 25 patients	Bilateral sagittal split mandibular ramus osteotomy	(1) Dental impact on daily living: DIDL (2) OHIP-14
(28) Alanko et al. [2]	Prospective	Finland	Initial sample: 60 patients Final sample: 22 patients	Bilateral sagittal osteotomy, bimaxillary osteotomy, maxillary osteotomy	(1) OQLQ (2) Rosenberg Self-Esteem Scale (3) Acceptance and Action Questionnaire
(29) Pelo et al. [39]	Prospective	Italy	Total sample: 30 patients Surgery-first group: 15 patients Conventional surgery group: 15 patients	Le fort I osteotomy, mandibular bilateral sagittal split osteotomy	(1) OQLQ (2) OHIP-14
(30) Zingler et al. [40]	Prospective	Germany	9 patients	Maxillary osteotomy, mandibular osteotomy, bimaxillary osteotomy	(1) OQLQ (2) SOC-29

**Table 2:** Results on patient satisfaction after surgery (*n*=10).

Study design	Follow-up duration	Main results	References
(1) Retrospective (postoperative analysis) Prospective (preoperative analysis)	At least 9 months after surgery	Of the participants, 95% were satisfied with the results of treatment (very satisfied: 66.7%; moderately satisfied: 28.4%); 7.5% were dissatisfied with the results; and 76.5% stated that they would undergo the surgery again.	Cunningham et al. [16]
(2) Prospective	T0: 1 month before surgery T1: 1 year after surgery	The mean VAS score for patient satisfaction was 8.8 (88%); 86% of participants would undergo surgery again. All investigated life aspects improved after surgery: work, livelihood, interpersonal relationships, leisure, mental health, health and perspective on life.	Forssell et al. [17]
(3) Prospective	T0: 1 week before surgery T1: 2 to 8 months after surgery	69.2% of participants were satisfied with surgery, and 23.1% were very satisfied; none of the participants reported dissatisfaction.	Bertolini et al. [18]
(4) Retrospective	Evaluation at 1, 2 and more than 2 years	Of the patients, 92% were satisfied, and 89% were aware of what to expect after discharge. Negative surgery-related outcomes tended to decrease along the follow-up.	Busby et al. [19]
(5) Prospective	Before surgery 6 months after surgery	Significant difference in satisfaction before (79.22 ± 18.42) and after (87.56 ± 15.50) (*p* < 0.01) surgery.	Murphy et al. [4]
(6) Retrospective	2.54 years after surgery	Participants reported satisfaction with the appearance of their face after treatment; smile, self-confidence (85.3%), social life (46%), eating (60.6%), and speech (39.3%).	Khattak et al. [24]
(7) Retrospective	T0: baseline T1: before orthodontic treatment T2: 8 weeks after surgery T3: 1 year after surgery T4: 10–14 years after surgery	Of the participants, 36% reported that they were very satisfied, 53% were moderately satisfied, and 8% were dissatisfied.	Trovik et al. [26]
(8) Prospective	T0: before surgery T1: after surgery	The scores on the FACE-Q used to assess satisfaction showed a significant increase of patient satisfaction after orthognathic surgery for the domains facial appearance overall (T0: 48.2 ± 3.2; T1: 72.9 ± 3.3), lower face and jawline (T0: 42.6 ± 6.3; T1: 83.3 ± 5.9) and all four chin items (*p* < 0.01).	Schwitzer et al. [29]
(9) Retrospective	At least 6 months after surgery	The mean SAT score was 23.9 ± 3.83; 95% of participants would undergo surgery again. The mean SAT score was higher for the participants subjected to sagittal osteotomy compared to that for the patients undergoing bimaxillary osteotomy (*p* < 0.05).	Bogusiak et al. [37]
(10) Prospective	T1: before treatment T2: 1 month after surgery T3: 6 months after treatment T4: 12 months after treatment T5: 18 after treatment T6: after the end of orthodontic-surgical treatment	Satisfaction was substantially lower for the group subjected to the surgery-first approach, but the difference was statistically nonsignificant compared to that of the conventional treatment group.	Huang et al. [38]

**Table 3:** Results for the OQLQ global and domain scores (*n*=12).

Study design	Follow-up duration	Main results	References
(1) Prospective	T0: baseline T1: 6 weeks after surgery T2: 6 months after surgery	T0-T1: no significant difference in the global score; decrease in the score for the domain facial esthetics. T0-T2: significant reductions in the global score and scores for 3 of 4 domains (social, facial esthetics, and oral function).	Lee et al. [9]
(2) Retrospective	21 months after surgery	Significant differences in the global score and all 4 domain scores between the pre- and postsurgery groups. However, no difference in the scores was found among the controls, postsurgery group, and patients who declined surgery.	Al-Ahmad et al. [20]
(3) Prospective	T0: baseline T1: 6 weeks after surgery T2: 6 months after surgery T3: after orthodontic treatment (at least 12 months after orthognathic surgery and 6 months after the end of orthodontic treatment)	T0-T1: significant reduction in the global score and scores for the domains social aspects and facial esthetics. T0–T2: significant reduction in the global score and scores for the domains social aspects, facial esthetics and oral function. T0–T3: significant reduction in the global score and all 4 domain scores.	Choi et al. [3]
(4) Prospective	T0: before surgery T1: 6 to 8 months after surgery	Significant reduction in OQLQ scores after surgery in both groups. At T0, a significant difference was found for the domains oral function and facial esthetics (*p* < 0.01) between groups. At T1, only the domain oral function exhibited a significant difference between the groups.	Khadka et al. [23]
(5) Prospective	T0: during orthodontic treatment T1: before surgery T2: 6 months after surgery	Significant differences in all OQLQ domains before and after surgery. Domains: esthetics (T1: 12.21 ± 5.87; T2: 7.00 ± 5.64); awareness (T1: 6.90 ± 4.80; T2: 5.73 ± 4.19); social (T0: 10.42 ± 8.33; T1: 5.73 ± 4.19); and function (T0: 7.46 ± 5.99; T1: 5.69 ± 5.77) (*p* < 0.05).	Murphy et al. [4]
(6) Retrospective	T0: before surgery T1: 2 years after surgery	Significant reductions in the global score (T0: 28/T1: 13.51) and all 4 domain scores (*p* < 0.01).	Wee and Poon [28]
(7) Retrospective	T0: before surgery T1: at least 1 year after surgery	Reduction in the OQLQ global score after surgery.	Abdullah [31]
(8) Prospective	T0: first visit T1: before surgery T2: 3 months after surgery T3: removal of orthodontic appliance	Conventional surgery group: significantly higher scores before surgery (T0: 53.87 ± 17.81; T1: 58.07 ± 18.18; *p* < 0.05). Significant reductions in the global score at T2 (23.53 ± 9.28) and T3 (11.60 ± 8.20) and in all 4 domain scores. Surgery-first group: reduction in the global score at T2 (23.09 ± 22.14) and T3 (11.36 ± 14.15) compared to that at T0 (51.64 ± 19.27). No significant difference between groups.	Park et al. [32]
(9) Prospective	T0: before surgery T1: 6 weeks after surgery T2: 6 months after surgery	Significant reduction in the OQLQ score at T1 (30.5 ± 19.5) and T2 (26.1.±19.3) compared to that of the controls. The domain facial esthetics exhibited the greatest variation before and after surgery (T0: 10.6 ± 6.0; T1: 5.5 ± 5.4; T2: 4.8 ± 5.0; *p* < 0.001).	Silva et al. [35]
(10) Prospective	Orthognathic surgery group: T0: before treatment; T1: after orthodontic assessment; T2–T4: during orthodontic treatment; T5: 1 year after surgery Control group: T0: before treatment; T1: 2 years after first examination; T2: 4 years after first examination	The global score and the score for the domain oral function increased at T2 (35.89 ± 23.39) compared to those at T0 (31.38 ± 20.71) (*p* < 0.001). The global score and all 4 domain scores decreased at T5 compared to those at T2 (*p* < 0.001).	Alanko et al. [2]
(11) Prospective	T0: before bracket placement T1: 1 month before surgery T2: 1 month after surgery	No significant difference between groups at T0 (surgery-first: 57 ± 10/conventional: 52 ± 10) or T2 (surgery-first: 22 ± 3/conventional: 29 ± 9). Poorer score at T1 (60 ± 9) for the conventional surgery group. Significant differences among T0, T1 and T2 in both groups (*p* < 0.05).	Pelo et al. [39]
(12) Prospective	T0: before surgery T1: 3 months after surgery	Significant reduction in the score at T1 (18 ± 12.69) compared to that at T0 (36 ± 17.24) (*p* < 0.015). The domains facial esthetics (*p*=0.022), oral function (*p*=0.051) and social aspects (*p*=0.057) were the most affected.	Zingler et al. [40]

**Table 4:** Results for the global and domain scores on the OHIP-14 (*n*=12).

Study design	Follow-up duration	Main results	References
(1) Prospective	T0: baseline T1: 6 weeks after surgery T2: 6 months after surgery	T0-T1: no significant difference in the global score 6 weeks after orthognathic surgery. However, a significant increase was observed for the score on the domain functional limitation and significant decreases were observed for the scores on the domains psychological discomfort and psychological disability. T0–T2: significant reductions in the global score and in all 7 domain scores.	Lee et al. [9]
(2) Prospective	T0: baseline T1: 6 weeks after surgery T2: 6 months after surgery T3: after orthodontic treatment (at least 12 months after orthognathic surgery and 6 months after the end of orthodontic treatment)	T0-T1: no significant changes in the global score. Significant reductions in the scores on the domains functional limitation and psychological discomfort 6 weeks after surgery. T0–T2: significant reductions in the global score and in all 7 domain scores. T0–T3: significant reductions in the global score and in all 7 domain scores.	Choi et al. [3]
(3) Prospective	12 months after surgery	Significant reductions in scores on the domains psychological discomfort, dissatisfaction with esthetics and social disability.	Rustemeyer et al. [22]
(4) Prospective	8.3 months after surgery	Significant reductions in scores on the domains psychological discomfort and social disability after surgery.	Rustemeyer and Gregersen [25]
(5) Prospective	T0: 1 week before surgery T1: 1 month after surgery T2: 3 months after surgery	Significant differences in all scores (global and domains) at all postsurgery time points; the scores increased 1 month after surgery and substantially decreased 3 months after surgery.	Corso et al. [30]
(6) Prospective	T0: before treatment T1: before surgery T2: 6 months after surgery T3: 12 months after removal of orthodontic appliance	Domain functional limitation: Class II: the global score increased at T1 (22.84 ± 3.40) compared to that at T0 (19.18 ± 2.97) but significantly decreased at T2 (8.64 ± 3.21) and T3 (6.87 ± 2.11) (*p* < 0.01). The scores on all the domains decreased at T2 and T3. Class III: reduction in the global score at T1 (17.63 ± 3.83), T2 (6.71 ± 2.45) and T3 (6.24 ± 2.66) compared to that at T0 (19.86 ± 2.57) (*p* < 0.01) The scores on all the domains decreased at T2 and T3. Differences between groups were significant at T1 (*p*=0.003) and T2 (*p*=0.008).	Baherimoghaddam et al. [33]
(7) Prospective	T0: before surgery T1: 6 weeks after surgery T2: 6 months after surgery	Significant reductions in OHIP-14 scores at T1 and T2 compared to those of the controls.	Silva et al. [35]
(8) Retrospective	T0: 1 month before surgery T1: 6 months after surgery	The scores for social disability, physical pain, psychological discomfort and dissatisfaction with esthetics significantly decreased after surgery in both groups. Bimaxillary osteotomy group: social disability T0: 0.94 ± 1.22, T1: 0.38 ± 0.81; physical pain T0: 1.17 ± 1.16, T1: 0.67 ± 0.72; psychological discomfort T0: 1.55 ± 1.03, T1: 1.19 ± 1.41; dissatisfaction with esthetics T0: 2.83 ± 1.13, T1: 1.89 ± 0.99. Bimaxillary osteotomy with genioplasty group: social disability T0: 1.33 ± 1.39, T1: 0.37 ± 0.53; physical pain T0: 1.29 ± 1.01, T1: 0.78 ± 0.75; psychological discomfort T0: 2.02 ± 1.05, T1: 0.74 ± 0.59; dissatisfaction with esthetics T0: 2.73 ± 1.14, T1: 0.41 ± 0.48. The domains psychological discomfort and dissatisfaction with esthetics exhibited significant differences favoring the bimaxillary osteotomy with genioplasty group.	Rustemeyer and Lehmann [27]
(9) Prospective	T0: before surgery T1: 4–6 months after surgery	Significant reduction in the global score from T0 (13.23 ± 6.45) to T1 (3.26 ± 4.19) (*p* < 0.001). Significant reductions in all domain scores at T1 (*p* < 0.001).	Göelzer et al. [5]
(10) Retrospective	T0: before surgery T1: 2 years after surgery	Significant reductions in the global score (T0: 14/T1: 4.68) and all domain scores (*p* < 0.01).	Wee and Poon [28]
(11) Prospective	T1: before surgery T2: 1 month after surgery T3: 6 months after treatment T4: 12 months after treatment T5: 18 months after treatment T6: after the end of orthodontic-surgical treatment	The quality of life of the surgery-first group significantly increased at T2 compared to that at T1, but no difference was found from T4 to T6. In the group subjected to conventional treatment, quality of life declined until T3 but in a nonsignificant manner; then, it significantly improved (*p* < 0.001).	Huang et al. [38]
(12) Prospective	T0: before bracket placement T1: 1 month before surgery T2: 1 month after surgery	No significant difference was found between groups at T0 (surgery-first: 16 ± 6/conventional: 13 ± 5) or T2 (surgery-first: 2 ± 1/conventional: 3 ± 1). Significant differences were found in each group among T0, T1 and T2 (*p* < 0.01). A significant difference was found in the conventional treatment group between T0 and T1 (*p*=0.05).	Pelo et al. [39]

**Table 5:** Results for the global and domain scores on the SF-36 (*n*=4).

Study design	Follow-up duration	Main results	References
(1) Prospective	T0: baseline T1: 6 weeks after surgery T2: 6 months after surgery	T0-T1: significant reductions in scores for the physical health, mental health, and social domains. T0–T2: no significant change in any domain, except for an increase in the score for the emotional component.	Lee et al. [9]
(2) Retrospective	21 months after surgery	Significant differences in the components general health, vitality and mental health between the pre- and postsurgery groups favoring the group of patients who underwent surgery.	Al-Ahmad et al. [20]
(3) Prospective	T0: baseline T1: 6 weeks after surgery T2: 6 months after surgery T3: after orthodontic treatment (at least 12 months after orthognathic surgery and 6 months after the end of orthodontic treatment)	T0-T1: significant reduction in the score for the domain physical health. T0–T3: significant improvement in the component mental health after the end of orthodontic treatment.	Choi et al. [3]
(4) Prospective	T0: before surgery T1: 6 to 8 months after surgery	Significant differences in the physical and bodily pain components (*p* < 0.05) between the groups at T0. No significant difference between groups at T1 (*p* > 0.05).	Khadka et al. [23]

**Table 6:** Methodological evaluation of the selected studies according to the Methodological Index for Non-Randomized Studies (MINORS). Scores were assigned as follows: 0 (not reported); 1 (reported but inadequate); and 2 (reported and adequate). The ideal global score is 16 for noncomparative studies and 24 for comparative studies [11].

Criteria	Cunningham et al. [16]	Forssell et al. [17]	Bertolini et al. [18]	Busby et al. [19]	Lee et al. [9]	Al-Ahmad et al. [20]	Choi et al. [3]	Silva et al. [21]	Rustemeyer et al. [22]	Khadka et al. [23]	Murphy et al. [4]	Khattak et al. [24]	Rustemeyer and Gregersen [25]	Trovik et al. [26]	Rustemeyer and Lehmann, [27]
A clearly stated aim	2	2	2	2	2	2	2	2	2	2	2	2	2	2	2
Inclusion of consecutive patients	1	2	2	2	2	1	2	1	1	2	2	1	2	1	0
Prospective collection of data	0	2	2	0	2	0	2	2	2	2	2	0	2	0	0
Endpoints appropriate for the study aim	1	1	1	1	2	2	2	1	2	2	2	2	2	2	2
Unbiased evaluation of endpoints	0	0	0	0	0	0	0	0	0	0	1	0	0	0	0
Appropriate follow-up	2	2	2	2	2	2	2	1	2	2	1	1	2	2	2
Loss to follow-up of less than 5%	0	2	0	0	2	0	0	0	0	0	0	0	0	0	0
Prospective sample size calculation	0	0	0	0	0	0	0	0	0	0	0	0	0	0	0
Adequate control group	NA	NA	NA	NA	NA	2	NA	NA	NA	1	NA	NA	NA	NA	2
Contemporary groups	NA	NA	NA	NA	NA	0	NA	NA	NA	2	NA	NA	NA	NA	2
Baseline equivalence of groups	NA	NA	NA	NA	NA	2	NA	NA	NA	2	NA	NA	NA	NA	2
Adequate statistical analyses	NA	NA	NA	NA	NA	2	NA	NA	NA	2	NA	NA	NA	NA	2
Total score	6	11	9	7	12	13	10	7	9	17	10	6	10	7	14

Criteria	Wee and Pon [28]	Göelzer et al. [5]	Schwitzer et al. [29]	Abdullah [31]	Corso et al. [30]	Park et al. [32]	Baherimoghaddam et al. [33]	Kilinc and Ertas [34]	Kurabe et al. [36]	Silva et al. [35]	Bogusiak et al. [37]	Huang et al. [38]	Alanko et al. [2]	Pelo et al. [39]	Zingler et al. [40]

A clearly stated aim	2	2	2	2	2	2	2	2	2	2	2	2	2	2	2
Inclusion of consecutive patients	1	2	1	1	1	1	2	2	2	2	2	2	1	1	2
Prospective collection of data	0	2	2	0	2	2	2	2	2	2	0	2	2	2	2
Endpoints appropriate for the study aim	2	2		2	2	2	2	2	2	2	1	1	2	2	2
Unbiased evaluation of endpoints	0	0	0	0	0	0	0	0	0	0	0	0	0	0	0
Appropriate follow-up	2	2	1	2	1	2	2	2	2	2	1	2	1	2	2
Loss to follow-up of less than 5%	0	0	0	0	0	0	0	0	0	2	0	0	0	0	0
Prospective sample size calculation	0	0	0	0	0	0	0	0	0	0	0	0	0	0	0
Adequate control group	NA	NA	NA	NA	0	2	2	2	NA	NA	NA	0	0	2	NA
Contemporary groups	NA	NA	NA	NA	1	2	2	2	NA	NA	NA	2	1	2	NA
Baseline equivalence of groups	NA	NA	NA	NA	2	2	2	2	NA	NA	NA	2	2	2	NA
Adequate statistical analyses	NA	NA	NA	NA	2	2	2	2	NA	NA	NA	2	2	2	NA
Total score	7	10	6	7	13	18	18	18	10	12	6	15	13	18	10

## References

[1] Soh C. L., Narayanan V. (2013). Quality of life assessment in patients with dentofacial deformity undergoing orthognathic surgery-a systematic review. *International Journal of Oral and Maxillofacial Surgery*.

[2] Alanko O., Tuomisto M. T., Peltomäki T., Tolvanen M., Soukka T., Svedström-Oristo A. L. (2017). A longitudinal study of changes in psychosocial well-being during orthognathic treatment. *International Journal of Oral and Maxillofacial Surgery*.

[3] Choi J.-Y., Song K.-G., Baek S.-H. (2009). Virtual model surgery and wafer fabrication for orthognathic surgery. *International Journal of Oral and Maxillofacial Surgery*.

[4] Murphy C., Kearns G., Sleeman D., Cronin M., Allen P. F. (2011). The clinical relevance of orthognathic surgery on quality of life. *International Journal of Oral and Maxillofacial Surgery*.

[5] Göelzer J. G., Becker O. E., Haas Junior O. L. (2014). Assessing change in quality of life using the oral health impact profile (OHIP) in patients with different dentofacial deformities undergoing orthognathic surgery: a before and after comparison. *International Journal of Oral and Maxillofacial Surgery*.

[6] Silva I., Suska F., Cardemil C., Rasmusson L. (2013). Stability after maxillary segmentation for correction of anterior open bite: a cohort study of 33 cases. *Journal of Cranio-Maxillofacial Surgery*.

[7] World Health Organization (1993). *WHOQOL: Study Protocol, MNH/PSF/93.9*.

[8] Lohr K. N., Aaronson N. K., Alonso J. (1996). Evaluating quality-of-life and health status instruments: development of scientific review criteria. *Clinical Therapeutics*.

[9] Lee L.-W., Chen S.-H., Yu C.-C., Lo L.-J., Lee S.-R., Chen Y.-R. (2007). Stigma, body image, and quality of life in women seeking orthognathic surgery. *Plastic and Reconstructive Surgery*.

[10] Kiyak H. A., McNeill R. W., West R. A., Hohl T., Bucher F., Sherrick P. (1982). Predicting psychologic responses to orthognathic surgery. *Journal of Oral and Maxillofacial Surgery*.

[11] Ryan F. S., Barnard M., Cunningham S. J. (2012). Impact of dentofacial deformity and motivation for treatment: a qualitative study. *American Journal of Orthodontics and Dentofacial Orthopedics*.

[12] Chen B., Zhang Z. K., Wang X. (2002). Factors influencing postoperative satisfaction of orthognathic surgery patients. *International Journal of Adult Orthodontics and Orthognathic Surgery*.

[13] Liberati A., Altman D. G., Tetzlaff J. (2009). The PRISMA statement for reporting systematic reviews and analyses of studies that evaluate health care interventions: explanation and elaboration. *Journal of Clinical Epidemiology*.

[14] Slim K., Nini E., Forestier D., Kwiatkowski F., Panis Y., Chipponi J. (2003). Methodological index for non-randomized studies (MINORS): development and validation of a new instrument. *ANZ Journal of Surgery*.

[15] Higgins J. P. T., Green S. (2011). *Cochrane Handbook for Systematic Reviews of Interventions Version 5.1.0*.

[16] Cunningham S. J., Hunt N. P., Feinmann C. (1996). Perceptions of outcome following orthognathic surgery. *British Journal of Oral and Maxillofacial Surgery*.

[17] Forssell H., Finne K., Forssell K., Panula K., Blinnikka L. M. (1998). Expectations and perceptions regarding treatment: a prospective study of patients undergoing orthognathic surgery. *International Journal of Adult Orthodontics and Orthognathic Surgery*.

[18] Bertolini F., Russo V., Sansebastiano G. (2000). Pre- and postsurgical psycho-emotional aspects of the orthognathic surgery patient. *International Journal of Adult Orthodontics and Orthognathic Surgery*.

[19] Busby B. R., Bailey L. J., Proffit W. R., Phillips C., White R. P. (2002). Long-term stability of surgical class III treatment: a study of 5-year postsurgical results. *International Journal of Adult Orthodontics and Orthognathic Surgery*.

[20] Al-Ahmad H. T., Al-Sa’di W. S., Al-Omari I. K., Al-Bitar Z. B. (2009). Condition-specific quality of life in Jordanian patients with dentofacial deformities: a comparison of generic and disease-specific measures. *Oral Surgery, Oral Medicine, Oral Pathology, Oral Radiology, and Endodontology*.

[21] Silva A. C. A. E., Carvalho R. A. S., Santos T. D. S., Rocha N. S., Gomes A. C. A., Silva E. D. D. O. E. (2013). Evaluation of life quality of patients submitted to orthognathic surgery. *Dental Press Journal of Orthodontics*.

[22] Rustemeyer J., Martin A., Gregersen J. (2012). Changes in quality of life and their relation to cephalometric changes in orthognathic surgery patients. *The Angle Orthodontist*.

[23] Khadka A., Liu Y., Li J. (2011). Changes in quality of life after orthognathic surgery: a comparison based on the involvement of the occlusion. *Oral Surgery, Oral Medicine, Oral Pathology, Oral Radiology, and Endodontology*.

[24] Khattak Z. G., Benington P. C. M., Khambay B. S., Green L., Walker F., Ayoub A. F. (2012). An assessment of the quality of care provided to orthognathic surgery patients through a multidisciplinary clinic. *Journal of Cranio-Maxillofacial Surgery*.

[25] Rustemeyer J., Gregersen J. (2012). Quality of Life in orthognathic surgery patients: post-surgical improvements in aesthetics and self-confidence. *Journal of Cranio-Maxillofacial Surgery*.

[26] Trovik T. A., Wisth P. J., Tornes K., Bøe O. E., Moen K. (2012). Patients’ perceptions of improvements after bilateral sagittal split osteotomy advancement surgery: 10 to 14 years of follow-up. *American Journal of Orthodontics and Dentofacial Orthopedics*.

[27] Rustemeyer J., Lehmann A. (2012). Reduction genioplasty enhances quality of life in female patients with prognathism and maxillary hypoplasia undergoing bimaxillary osteotomy. *International Journal of Oral and Maxillofacial Surgery*.

[28] Wee T. H., Poon C. Y. (2014). Quality of life treatment outcomes of class III skeletal patients after bimaxillary osteotomies. *Proceedings of Singapore Healthcare*.

[29] Schwitzer J. A., Albino F. P., Mathis R. K., Scott A. M., Gamble L., Baker S. B. (2015). Assessing patient-reported outcomes following orthognathic surgery and osseous genioplasty. *Journal of Craniofacial Surgery*.

[30] Corso P. F., Oliveira F. A., Costa D. J., Kluppel L. E., Rebellato N. L., Scariot R. (2016). Evaluation of the impact of orthognathic surgery on quality of life. *Brazilian Oral Research*.

[31] Abdullah W. A. (2015). Changes in quality of life after orthognathic surgery in Saudi patients. *The Saudi Dental Journal*.

[32] Park J.-K., Choi J.-Y., Yang I.-H., Baek S.-H. (2015). Patientʼs satisfaction in skeletal class III cases treated with two-jaw surgery using orthognathic quality of life questionnaire. *Journal of Craniofacial Surgery*.

[33] Baherimoghaddam T., Tabrizi R., Naseri N., Pouzesh A., Oshagh M., Torkan S. (2016). Assessment of the changes in quality of life of patients with class II and III deformities during and after orthodontic-surgical treatment. *International Journal of Oral and Maxillofacial Surgery*.

[34] Kilinc A., Ertas U. (2015). An assessment of the quality of life of patients with class III deformities treated with orthognathic surgery. *Journal of Oral and Maxillofacial Surgery*.

[35] Silva I., Cardemil C., Kashani H. (2016). Quality of life in patients undergoing orthognathic surgery—a two-centered Swedish study. *Journal of Cranio-Maxillofacial Surgery*.

[36] Kurabe K., Kojima T., Kato Y., Saito I., Kobayashi T. (2016). Impact of orthognathic surgery on oral health-related quality of life in patients with jaw deformities. *International Journal of Oral and Maxillofacial Surgery*.

[37] Bogusiak K., Kowalczyk T., Arkuszewski P. (2016). Satisfaction with life in patients with skeletal class III malocclusion after orthognathic surgery. *Dental and Medical Problems*.

[38] Huang S., Chen W., Ni Z., Zhou Y. (2016). The changes of oral health-related quality of life and satisfaction after surgery-first orthognathic approach: a longitudinal prospective study. *Head and Face Medicine*.

[39] Pelo S., Gasparini G., Garagiola U. (2017). Surgery-first orthognathic approach vs traditional orthognathic approach: oral health-related quality of life assessed with 2 questionnaires. *American Journal of Orthodontics and Dentofacial Orthopedics*.

[40] Zingler S., Hakim E., Finke D. (2017). Surgery-first approach in orthognathic surgery: psychological and biological aspects—a prospective cohort study. *Journal of Cranio-Maxillofacial Surgery*.

[41] Patrick D., Bergner M. (1990). Measurement of health status in the 1990s. *Annual Review of Public Health*.

[42] Slade G. D., Spencer A. J. (1994). Development and evaluation of the oral health impact profile. *Community Dental Health*.

[43] Kiyak H. A. (1993). Psychological aspects of orthognathic surgery. *Psychology & Health*.

[44] Helm S., Kreiborg S., Solow B. (1985). Psychosocial implications of malocclusion: a 15-year follow-up study in 30-year-old danes. *American Journal of Orthodontics*.

